# Results of Radiation Therapy as Local Ablative Therapy for Oligometastatic Non-Small Cell Lung Cancer

**DOI:** 10.3390/cancers13225773

**Published:** 2021-11-18

**Authors:** David L. Billing, Andreas Rimner

**Affiliations:** Department of Radiation Oncology, Memorial Sloan Kettering Cancer Center, New York, NY 10065, USA; billingd@mskcc.org

**Keywords:** oligometastatic, non-small cell lung cancer, radiotherapy, stereotactic body radiotherapy, local ablative therapy

## Abstract

**Simple Summary:**

Metastatic lung cancer represents a heterogeneous population of patients. Up to half of patients with metastatic lung cancer may be oligometastatic, an intermediate step between localized disease and widespread metastasis that is characterized by a limited number of metastatic deposits. Patients with oligometastatic lung cancer may benefit from local ablative therapy (LAT) directed at metastatic deposits in addition to standard-of-care systemic therapy. Radiation therapy is one option for LAT in lung cancer. This review will discuss the studies that have established the safety and efficacy of radiation therapy for oligometastatic lung cancer.

**Abstract:**

Oligometastatic cancer is characterized by a limited number of metastatic deposits. Compared with lung cancer patients who have more widespread disease, oligometastatic lung cancer patients have more favorable survival outcomes. Therefore, it has been hypothesized that local ablative therapy (LAT) directed at the metastatic deposits in addition to standard-of-care systemic therapy may further improve survival outcomes in oligometastatic lung cancer patients. One LAT modality that has been utilized in oligometastatic lung cancer is radiation therapy. In particular, ultra-hypofractionated radiotherapy, also known as stereotactic body radiotherapy (SBRT), has been shown to provide excellent local control with a favorable safety profile. Here, we reviewed the retrospective studies and prospective trials that have deployed radiation therapy as LAT in oligometastatic lung cancer, including randomized studies showing benefits for progression-free survival and overall survival with the addition of LAT. We also discuss the impact of targeted therapies and immunotherapy on radiation as LAT.

## 1. Introduction

Lung cancer accounts for approximately 12% of cancer diagnoses yet comprises 22% of cancer deaths annually in the United States [1]. Nearly half of all lung cancer cases are metastatic, i.e., Stage IV by AJCC staging at diagnosis. While metastatic lung cancer has been traditionally associated with uniformly poor survival, in the preceding decades, it has become apparent that metastatic lung cancer represents a heterogeneous population with differing outcomes based on the extent and location of metastatic deposits. The concept of an oligometastatic state, an intermediate step between localized disease and widespread dissemination that is characterized by a limited number of metastatic deposits and possibly associated with more favorable outcomes, was first proposed by Hellman and Weichselbaum in 1995 [2]. While many definitions of what constitutes an oligometastatic state have been utilized over the years with respect to lung cancer, it is generally defined as disease with ≤5 extrathoracic metastatic lesions in ≤3 organs [3]. Furthermore, oligometastatic disease can also be synchronous (i.e., distant disease present at the time of diagnosis) or metachronous, in that distant disease becomes clinically apparent after definitive treatment of the localized disease, also sometimes referred to as “oligorecurrent”. Patients with metachronous oligometastatic disease have been observed to have more favorable outcomes than those with synchronous oligometastatic disease [4,5]. However, the majority of studies conducted in oligometastatic lung cancer have focused on synchronous oligometastatic disease. Additionally, metastatic disease may be oligoprogressive, meaning that a limited number of metastatic sites progress while most other sites are stable or respond to systemic therapy. Oligoprogressive lung cancer, which is beyond the scope of this review, typically occurs in the setting of polymetastatic disease and has been discussed elsewhere [6,7,8].

While differing definitions of “oligometastatic” complicate efforts to capture the incidence of this disease state, it is estimated that oligometastatic lung cancer may represent up to 25–50% of all metastatic lung cancer cases [9,10]. Furthermore, advances in imaging technology and more widespread use of modalities such as brain MRI and PET/CT have allowed for more accurate staging of lung cancer patients and detection of metastases that would have previously been subclinical. These oligometastatic lung cancer patients have been observed to have more favorable survival outcomes compared with patients with more widely metastatic disease [9,10,11]. This observed heterogeneity of outcomes within patients with metastatic lung cancer is beginning to be reflected in lung cancer staging. At present, the AJCC (8th edition) stages patients with a single extra thoracic metastasis as M1b and Stage IVA, compared with patients who have more widespread metastases who are classified as M1c and Stage IVB [12].

Given the more favorable survival outcomes observed in oligometastatic lung cancer patients, it has been hypothesized that these patients may benefit from a more aggressive treatment approach with metastasis-directed local therapy in addition to systemic therapy. Advances in targeted systemic therapies for metastatic NSCLC such as EGFR inhibition and immunotherapy have improved survival outcomes, increasing the importance of long-term local control of metastatic deposits. Patterns of failure analyses suggest that the most likely locations of failure after first-line chemotherapy are the initially involved sites [13], providing further rationale for metastasis-directed therapy. Locally aggressive therapy or locally ablative therapies (LAT) that have been utilized in the oligometastatic state include surgery, ionizing radiation therapy and ablative techniques, e.g., radiofrequency or microwave ablation, among others. The goal of LAT has to be the complete eradication of viable tumor cells in the treated site for durable local control. Whenever possible, LAT should aim to treat all oligometastatic sites of disease in order to render patients free from gross/radiographically apparent residual disease. Radiation therapy, which is the focus of this review article, has emerged as a particularly efficacious local therapy, with multiple retrospective and prospective studies showing both local control and survival benefits. As many lung cancer patients have significant medical comorbidities, radiation has the benefit of being non-invasive compared with other LAT modalities and therefore is a treatment option for most patients. Additionally, not all metastatic sites may exist in locations that are amenable to surgical intervention, whereas most are able to be treated with radiation irrespective of the site.

Recent technological advances in multiple aspects of radiotherapy techniques have allowed for more accurate delivery of radiation, thus permitting smaller treatment margins and minimization of the dose to the surrounding tissues, thereby limiting healthy organ toxicity. In particular, radiation planning using three-dimensional imaging techniques, such as computed tomography (CT) or magnetic resonance imaging (MRI), has allowed for more accurate target and normal organ definition, as well as a better understanding of the relationship between radiation dose and normal organ toxicity [14]. As radiation therapy is often administered over the course of several treatments or fractions, precise and reproducible delivery of radiotherapy between these fractions is heavily dependent on the quality of image guidance to verify the radiation target and normal organ positioning (also known as interfractional monitoring). The development and use of the cone beam CT as on-board imaging for the linear accelerator has significantly improved the quality of image guidance in recent decades [15,16]. In addition, the ability to account for radiation target movement in areas of the body with significant motion, such as the lung or the abdomen, has been refined by the use of techniques such as deep inspiratory breath holding and the four-dimensional CT scan [17,18], as well as by improved target tracking within each treatment (i.e., intrafractional monitoring) [19]. Finally, radiation delivery using intensity modulated radiotherapy (IMRT) has significantly improved the conformality of radiation dose to the target, which is of particular importance when the radiation target abuts critical normal organs such as the spinal cord.

Historically, radiation therapy was often delivered in 1.8–2 Gy fractions over the course of several weeks (i.e., conventionally fractionated radiation). While conventionally fractionated radiation is still used to good effect in many different disease sites, including the definitive treatment of locally advanced NSCLC, the technological advances described above have allowed for the safe and accurate delivery of higher doses per fraction. Ultra-hypofractionated radiation, also referred to as stereotactic body radiotherapy (SBRT), stereotactic ablative radiotherapy (SABR) and stereotactic radiosurgery (SRS), is a form of image-guided radiation that utilizes high doses of radiation per fraction (>5 Gy per fraction), frequently delivered over one to five fractions. From a radiobiology standpoint, a higher dose per fraction allows for the delivery of a higher biological effective dose (BED) to the tumor, increasing the chance of local control through complete eradication of viable tumor cells. Indeed, the relationship between higher BED and improved local control was demonstrated in a retrospective analysis of clinical trials using SBRT for early-stage NSCLC [20]. Another large retrospective review from the Memorial Sloan Kettering Cancer Center in patients with Stage I NSCLC showed improved local control and overall survival with SBRT compared with conventionally fractionated radiation courses [21]. The efficacy of SBRT has also been demonstrated in the metastatic setting in regions other than the lung. Notably, retrospective studies from the Memorial Sloan Kettering Cancer Center of single-fraction SRS for metastatic disease to the spine have demonstrated excellent local control with minimal high-grade toxicity, irrespective of histology [22,23]. Furthermore, a recently published Phase III trial of single-fraction SRS vs. three-fraction SBRT for oligometastatic disease (including 10% lung cancer patients) showed superior local control and a decreased incidence of distant metastatic progression with single-fraction SRS [24].

In addition to the benefits towards tumor control described above, SBRT is often more convenient for the patient, as it reduces the number of treatments necessary for a given radiation course, lessens the financial burden associated with long radiation courses, and decreases the demand on healthcare resources. Taken together, SBRT has become a favored radiation-based approach with respect to LAT in oligometastatic lung cancer because of its high efficacy, with consistent reports of durable long-term local control of ≥90%, as well as its excellent toxicity profile in an otherwise frequently frail patient population. The focus of this review was the use of radiation-based approaches to LAT, most commonly SBRT, in the treatment of oligometastatic lung cancer.

## 2. Retrospective Studies

Several early retrospective studies provided the rationale for future prospective studies of radiation as LAT in oligometastatic NSCLC. One early retrospective study looked at 23 patients with oligometastatic NSCLC, defined as one or two metastatic sites, who received definitive treatment of the primary tumor (chemotherapy with surgery and/or radiation) [25]. Most patients in this series (15) received RT alone as LAT, with the brain as the most common site of metastasis. Median overall survival (OS) was observed to be 20 months, including five patients surviving beyond 36 months. These survival outcomes compared favorably with unselected Stage IV NSCLC patients and were noted to be most similar to those of Stage III patients, suggesting a benefit of LAT. Similarly, another single-institution retrospective review investigated the survival outcomes of 74 patients with oligometastatic NSCLC, of whom 44 patients were treated with LAT [26]. Radiation was the most commonly used LAT, accounting for 35 of the 44 cases. Receipt of LAT was associated with improved overall survival (OS) in univariate analysis but was not significant in multivariate analysis. Performance status, volume of the primary tumor and receipt of at least 63 Gy to the primary tumor were associated with improved OS in multivariate analysis. This observation of favorable OS outcomes with aggressive treatment of the primary site (with chemotherapy plus surgery and/or radiation) has also been observed in a metanalysis of 668 oligometastatic NSCLC patients [27], a secondary analysis of two prospective trials involving 274 oligometastatic and Stage IV non-oligometastatic NSCLC patients [28], and a large retrospective review from the Memorial Sloan Kettering Cancer Center [29]. These studies align with an earlier study noting that the most common site of recurrence in metastatic NSCLC is at the location of the primary tumor [30], suggesting the importance of definitive therapy targeting the primary tumor in combination with LAT. 

One of the largest retrospective analyses of SBRT as LAT in oligometastatic disease included 309 patients, of whom approximately one-third were patients with lung cancer, and showed a median OS of 24 months [31]. The authors also identified the factors associated with worse OS, which included non-adenocarcinoma histology and the presence of intracranial metastases. Another single-institution retrospective propensity score-matched analysis of 90 patients with oligometastatic NSCLC (one to three metastatic sites) who received at least two cycles of consolidative chemotherapy without disease progression compared patients who received LAT with those who did not [32]. Once again, radiation, particularly SBRT, was given as the primary LAT in the majority of patients. Their analysis showed a significant improvement in outcomes with receipt of LAT in terms of both progression-free survival (PFS) (11.3 vs. 8.0 months) and OS (27.1 vs. 13.1 months). Taken together, these retrospective studies suggesting OS and PFS benefits with radiation as LAT provided the rationale for the development of prospective studies in oligometastatic lung cancer.

## 3. Single-Arm Prospective Studies

Building upon the retrospective studies described above, many single-arm prospective studies have investigated the safety and survival outcomes of radiation in oligometastatic lung cancer (see Table 1). One of the earliest prospective Phase I/II trials of SBRT as LAT focused on patients with up to three hepatic metastases [33]. In this trial, 47 patients, including 10 patients with lung cancer, were treated with an SBRT regimen of 60 Gy in three fractions to the liver metastases. An excellent 2-year local control rate of 92% was observed, and only a single patient experienced Grade 3+ toxicity. While about half of the patients had active extrahepatic disease and therefore may not have been technically oligometastatic, this trial helped to establish SBRT as a feasible LAT. 

Subsequent prospective trials expanded the use of consolidative radiation to sites other than the liver. A single-institution dose escalation trial at the University of Chicago included 61 patients with one to five sites of extracranial oligometastatic disease who were given SBRT to all known metastatic deposits in order to evaluate the safety and efficacy of SBRT [34]. While this trial enrolled multiple cancer types, there was a plurality of patients with lung cancer (11 NSCLC and five SCLC). The maximal tolerated dose was not reached, as there were no dose-limiting toxicities in any of the cohorts, including the highest dose cohort (48 Gy in three fractions). Only 11.3% of patients experienced Grade 3 toxicity. Local control rates at 1 and 2 years were 67.2% and 52.7%, respectively, whereas 1- and 2-year PFS was noted to be 33.3% and 22.0%. Overall survival was also noted to be favorable, with rates of 81.5% at 1 year and 56.7% at 2 years. While some of the lower-dose cohorts utilized suboptimal treatment regimens by modern standards, this trial demonstrated the feasibility of targeting all known sites of oligometastatic disease with SBRT, as well as highlighting the favorable survival outcomes in comparison with unselected metastatic patients.

Similarly, Collen et al. conducted a Phase II study of SBRT that included only patients with oligometastatic NSCLC at Universitair Zieknhuis Brussel [35]. In this trial, 26 patients with five or fewer metastases were treated 50 Gy in 10 fractions delivered to all sites of distant disease. In contrast to the studies above, brain metastases were included in this trial. With a median follow-up of 16.4 months, local control was 85%, 1-year PFS was 45% and 1-year OS was 67%, with only two patients experiencing Grade 3 toxicity. 

Radiation treatment approaches other than SBRT have also been investigated in prospective trials of LAT. In a Phase II trial of 39 patients with oligometastatic NSCLC, patients received LAT as either surgery or fractionated radiation to each metastasis, although most patients were treated with radiation [42,43]. The most commonly used fractionation scheme was 54 Gy in fractions of 1.8 Gy twice daily to metastases in the bone or adrenal gland. One-year PFS and OS were 51.3% and 56.4% respectively. A subsequent long-term update showed 5- and 6-year OS rates of 7.7% and 2.5%, with only three patients experiencing local recurrence. 

Systemic therapy is a cornerstone of treatment for metastatic lung cancer. As discussed above, aggressive treatment of the primary site has been associated with improved survival outcomes in retrospective analyses involving patients with oligometastatic lung cancer [26,27,28,29]. While the previously discussed initial trials did not select patients by the receipt, response or timing of systemic therapy, many more recent prospective studies have included these parameters in an effort to further stratify patients who may benefit from LAT. In particular, one Phase II study looked at oligometastatic NSCLC patients who progressed after first-line platinum-based chemotherapy [36]. Patients were eligible if they had six or fewer extracranial metastases and were treated with SBRT delivered to all sites of disease in combination with erlotinib, an EGFR inhibitor. Despite the use of an EGFR inhibitor, testing for an EGFR mutation was not required in this trial. Of the 24 patients enrolled in the trial, 13 underwent testing and no EGFR mutations were detected. With a median follow-up of 11.6 months, the median PFS and OS were 14.7 months and 20.4 months, respectively—relatively good outcomes in this population with a poor response to initial chemotherapy and likely ineffective second-line systemic treatment with erlotinib. Local control was excellent, with only three patients experiencing progression of a lesion targeted by SBRT. As observed in previous trials, serious toxicity was rare, with only two patients experiencing a Grade 3 event.

In contrast to the previous trial, Petty et al. enrolled oligometastatic NSCLC patients who achieved a partial response or had stable disease after first-line platinum-based chemotherapy [44]. The patients then received SBRT applied to all metastatic sites, as well as conventionally fractionated radiation applied to the primary tumor. While the trial closed early due to poor accrual, the median PFS and OS were promising at 11.2 months and 28.4 months, respectively.

Another recent single-institution Phase II study enrolled 50 patients with synchronous oligometastatic NSCLC, defined as five or fewer sites of disease, including the primary tumor, and who had a complete response to induction chemotherapy in all extrathoracic metastases but residual intrathoracic disease [45]. These patients were then treated with SBRT targeting the residual intrathoracic disease with 45 to 60 Gy in three to five fractions. Approximately 40% of patients were treated with an EGFR inhibitor. The primary outcome of progression-free survival was a median of 34.3 months, and the median overall survival was not reached after a median follow-up of 19 months, outcomes that compared favorably with historical data despite the patients receiving no treatment directed at the extrathoracic metastases. Taken together, the previous three trials helped to establish SBRT as a feasible and safe treatment option for oligometastatic NSCLC with variable responses to first-line chemotherapy. 

While the trials above primarily focused on synchronous oligometastatic NSCLC, a recent large prospective registry-based study focused on metachronous oligometastatic cancer [46]. This study included 1422 patients, including 64 patients with lung cancer, who had three or fewer extracranial metastases and were treated with SBRT doses ranging from 24 Gy to 60 Gy in three to eight fractions. Overall survival at 1 and 2 years was 92.3% and 79.2%, respectively (80.2% and 65.4% for patients with lung cancer) without a single treatment-related death. Overall rates of Grade 3+ toxicity were low, with the most common Grade 3+ adverse event being fatigue in 2% of patients.

While data from these single-arm prospective studies provided evidence showing the safety and feasibility of radiation as LAT, they also demonstrated better than expected survival outcomes for patients with oligometastatic NSCLC. Given their non-randomized nature, however, there was concern that these improved outcomes were due to selection of a favorable subset of patients rather than the intervention itself. Furthermore, these studies were subject to immortal time bias, as patients who were selected for LAT to distant metastases must survive long enough to undergo LAT and were thus guaranteed to survive to this point, whereas the comparison patients had no such survival requirement [47,48]. Hence, randomized studies of radiation as LAT for oligometastatic lung cancer remain the standard for demonstrating efficacy.

## 4. Randomized Prospective Studies

Given the promise of earlier retrospective studies and single-arm prospective studies combined with the importance of randomized data in establishing LAT for oligometastatic lung cancer, it was disappointing that many early randomized prospective studies were closed early due to low accrual. As has been discussed elsewhere [48,49], this was likely due to a combination of factors, including a lack of equipoise amongst the enrolling physicians in the light of promising non-randomized prospective and retrospective data, overly stringent inclusion criteria, the availability of LAT off-trial, and patient unwillingness to accept randomization to no LAT. To date, three randomized prospective studies investigating LAT, primarily SBRT, for oligometastatic cancer have been published (see Table 1). 

The first published randomized multi-institutional trial of LAT for oligometastatic lung cancer was the landmark study by Gomez et al. [37]. This trial randomized patients with oligometastatic NSCLC after first-line systemic therapy to either LAT to all sites of active disease with or without maintenance systemic therapy or maintenance chemo/observation alone, and is described in greater detail in a separate review article in this Special Issue. Both surgery and radiation therapy were used as LAT, and the primary outcome of the study was progression-free survival, with a secondary outcome of overall survival.

While the trial initially planned to enroll 94 patients, the trial was stopped early, with 49 patients enrolled after a planned interim analysis showed a statistically significant benefit with LAT. The median PFS was 11.9 months in the LAT arm vs. 3.9 months in the control arm (*p* = 0.005). An update with long-term follow-up (median 38.8 months) showed a durable PFS benefit with LAT (median 14.2 months vs. 4.4 months, *p* = 0.022) [38]. While an OS benefit was not observed in the first report, with longer follow-up, an improvement in OS with LAT was observed (41.2 months vs. 17.0 months, *p* = 0.017). 

Another randomized Phase II single institution trial at the University of Texas Southwestern Medical Center enrolled 29 patients with oligometastatic NSCLC who did not progress after first-line chemotherapy and randomized them to SBRT targeting all sites of metastatic disease plus maintenance chemotherapy or maintenance chemotherapy alone [39]. In contrast to Gomez et al., patients could have up to five metastatic sites, patients with EGFR mutations were excluded from the trial and radiation was the only LAT administered. Additionally, patients with untreated brain metastases after systemic therapy were excluded, although treatment of brain metastases prior to chemotherapy was permitted. In the study population, the median number of disease sites was three and the most common site of metastasis was intrathoracic disease. Similar to that of Gomez et al., this trial was also closed through early accrual after an interim analysis showed a significant benefit in PFS in the SBRT arm. With a median follow-up of 9.6 months, PFS in the SBRT arm was 9.7 months vs. 3.5 months in the chemotherapy-alone arm (*p* = 0.01). Median overall survival was not reached in the SBRT arm compared with 17 months in the chemotherapy-alone arm, with the caveat that the trial was not powered to detect an overall survival difference. No Grade 3 or higher toxicities were noted as being attributable to SBRT. 

The most recently published randomized trial of radiation as LAT for oligometastatic cancer was the SABR-COMET trial [40,41]. This study was a multi-institutional Phase II trial that enrolled multiple different primary tumor types, including 18 patients with lung cancer out of a total of 99 participants. Patients were eligible if they had a controlled primary tumor that received definitive therapy at least 3 months prior to enrollment and up to five metastatic deposits amenable to SBRT. They were then randomized in a 2:1 allocation to SBRT applied to all metastatic sites with standard-of-care systemic therapy or standard-of-care systemic therapy with conventional palliative radiotherapy for symptomatic/at-risk metastases. Brain metastases were permitted but were present in only 4% of study participants, with the most common sites of metastases being the lung and bone. While up to five metastases were permitted, most patients in the study had three or fewer metastases, with only 7% of enrollees possessing four or more sites of disease. As had been detected in the previous trials, there was a significant benefit in PFS with LAT: after a median follow-up of 51 months, the median PFS in the SBRT arm was 11.6 months compared with 5.4 months in the control arm (*p* = 0.001). Additionally, there was also a significant OS benefit observed, with a median OS of 50 months in the SBRT arm compared with 28 months in the control arm (*p* = 0.006). In contrast to the previous trials, there were three treatment-related deaths in the SBRT arm (pneumonitis, pulmonary abscess and perforated gastric ulcer) compared with none in the control arm, although the rates of toxicity and severe toxicity were not statistically significant. 

Taken together, the three published randomized trials have provided significant evidence to support the use of radiation as LAT as a safe and effective treatment for oligometastatic NSCLC. In particular, all three trials have shown significant PFS benefits, and two of the three demonstrated an OS benefit. Furthermore, in each of the trials, there were low rates of serious adverse events that were comparable between SBRT and control arms. However, the data discussed above await confirmation in larger Phase III trials (see below), as the published trials enrolled relatively small numbers of patients. Several open questions still remain owing to the differing inclusion criteria and protocols of each trial, including the choice and timing of systemic therapy, management of thoracic nodal disease, and optimal selection of patients for LAT based on the number and location of metastatic deposits.

## 5. LAT Combined with Systemic Agents, Conclusions and Future Directions

In recent years, the systemic treatment of metastatic NSCLC has been revolutionized by the development of both targeted therapies such as EGFR inhibitors as well as immunotherapy, which have significantly improved survival outcomes. Given that these agents came into widespread use after the randomized trials discussed above, their impact on radiation as LAT for NSCLC is unclear. As originally hypothesized by Hellman and Weichselbaum, more effective systemic therapy would eliminate micrometastases or even induce an oligometastatic state that would make local control of macrometastatic deposits important for further improvements in long-term survival outcomes [2,50]. Indeed, the Gomez et al. trial supported the hypothesis of an induced oligometastatic state, as the trial enrolled patients with oligometastatic disease based on the number of metastatic deposits clinically apparent after receipt of systemic therapy [37]. 

Early data combining radiation and EGFR inhibitors for oligometastatic NSCLC are beginning to emerge. In one single-arm Phase II study, 16 patients harboring an EGFR-mutant NSCLC with four or fewer distant metastases were treated with 3 months of an EGFR inhibitor, followed by SBRT directed to all sites of disease [51]. Unfortunately, the trial was closed early due to slow accrual. With a median follow-up of 39.1 months, the 1-year PFS rate was noted to be 68.8% and median OS was 43.3 months, with no Grade 3 or higher radiation-related toxicity. The SINDAS trial (NCT02893332) is an ongoing randomized Phase III trial that has presented promising data in an interim analysis [52]. In this trial, patients with EGFR-mutant NSCLC with five or fewer metastases were randomized to EGFR inhibition alone or to SBRT to all metastatic sites, followed by EGFR inhibition. With 133 patients enrolled and a median follow-up of 19.6 months, significant improvements with SBRT were seen in the median PFS (20.2 vs. 12.5 months, *p* < 0.001) and median OS (25.5 vs. 17.4 months, *p* < 0.001) without significant differences in toxicity. While encouraging, these results await confirmation through full data maturation. Another ongoing study that will help to clarify the role of LAT in EGFR-mutant NSCLC is the NORTHSTAR trial (NCT03410043) which randomizes patients to osimertinib with or without LAT (radiation or surgery) for Stage IIIB and Stage IV NSCLC (see Table 2).

Immune checkpoint inhibitors improve survival in advanced NSCLC and are now the standard of care [53,54,55]. Given that immunotherapy was established after the three major published randomized studies of LAT in NSCLC, it is currently unclear what the impact will be on radiation for oligometastatic NSCLC. On one hand, radiation may potentiate the effect of immunotherapy through the abscopal effect, in essence introducing tumor-associated neoantigens, leading to a response in non-radiation targeted lesions as previously observed [56,57,58,59]. On the other hand, radiation combined with immunotherapy may lead to increased toxicity or decreased efficacy of the immunotherapy by damaging immune cells near the radiation target. At the time this article was written, the only published prospective trial of immunotherapy and radiation in oligometastatic NSCLC was a single-arm Phase II study [60]. In this study at the University of Pennsylvania, 51 patients with oligometastatic NSCLC (defined as four or fewer metastatic deposits) were treated with SBRT applied to metastatic deposits, followed by pembrolizumab 4 to 12 weeks after completion of the course of radiation. With a median follow-up of 25 months, the median PFS was observed to be 19.1 months, and the median overall survival was 41.6 months, both of which compare very favorably to historical controls, although an additional OS analysis is planned after further follow-up. Given the importance of immunotherapy in the treatment of advanced NSCLC, there are several ongoing clinical trials that combine radiation with immune checkpoint inhibitors. The CHESS trial (NCT03965468) is a Phase II study of upfront SBRT directed to oligometastatic deposits with chemoimmunotherapy followed by radiation and/or surgery of the primary tumor. Another Phase III trial is the LONESTAR study (NCT03391869) which randomizes patients with metastatic NSCLC to immunotherapy alone or immunotherapy followed by LAT (including radiation or surgery).

Finally, there are several ongoing trials that look to expand upon the previously published randomized studies. NRG LU-002 (NCT03137771) is a large national clinical trial in the United States that randomizes patients with oligometastatic NSCLC (defined as three or fewer extracranial metastases) to maintenance systemic therapy or maintenance systemic therapy with SBRT. Importantly, patients enrolling in this trial will be treated with first-line immunotherapy as is now the standard of care, and the trial will provide further insight on the role of LAT for oligometastatic NSCLC in the immunotherapy era. Similarly, the SARON study (NCT02417662) is a randomized Phase II/III trial in the United Kingdom for patients with five or fewer metastases. Other studies, such as SABR-COMET 10 (NCT03721341), look to expand upon the traditional limits of consolidative therapy for oligometastatic disease of five or fewer metastases to up to 10 lesions.

Taken together, these large randomized studies incorporating modern targeted and immunotherapies will help to further define the role of radiation in oligometastatic NSCLC, and to further identify the patients who will benefit from LAT.

## Figures and Tables

**Table 1:** Selected prospective trials of radiation as local ablative therapy for oligometastatic NSCLC.

Study (Year)	Design	N	Primary	Sites	LAT	Endpoints (Median)	Toxicity
Salama et al. (2012) [34]	Single-arm	61	Multiple (NSCLC = 11)	≤5	SBRT	PFS: 5.1 mo	Grade 3+: 11.7%
Collen et al. (2014) [35]	Single-arm	26	NSCLC	≤5	Hypofrac RT	PFS: 11.2 mo OS: 23 mo	Grade 3+: 8%
Iyengar et al. (2014) [36]	Single-arm	24	NSCLC	≤6	SBRT	PFS: 14.7 mo OS: 20.4 mo	Grade 3+: 8% Grade 5: 4%
Gomez et al. (2016, 2019) [37,38]	Randomized LAT vs. MCT	49	NSCLC	≤3	Surgery, SBRT, Hypofrac RT, Conventional RT	PFS: 11.9 vs. 4.4 mo MCT (*p* = 0.005) OS: 41.2 vs. 17.0 mo MCT (*p* = 0.017)	Grade 3: 20% vs. 8.3% MCT (NS) Grade 4+: 0%
Iyengar et al. (2018) [39]	Randomized LAT vs. MCT	29	NSCLC	≤5	SBRT	PFS: 9.7 vs. 3.5 mo MCT (*p* = 0.01) OS: not reached vs. 17 mo (NS)	Grade 3+: 0%
Palma et al. (2019, 2020) [40,41]	Randomized LAT vs. MCT	99	Multiple (NSCLC *n* = 18)	≤5	SBRT	PFS: 11.6 vs. 5.6 mo MCT (*p* = 0.001) OS: 50 vs. 28 mo MCT (*p* = 0.006)	Grade 2+: 29% Grade 5: 4.5%

LAT: local ablative therapy; Sites: number of lesions, SBRT: stereotactic body radiotherapy; PFS: progression-free survival; OS: overall survival; hypofract RT: hypofractionated radiotherapy; MCT: maintenance chemotherapy.

**Table 2:** Randomized trials of radiation as local ablative therapy for oligometastatic NSCLC in progress.

Study	Design	N	Primary	Sites	LAT	Primary Endpoint
SINDAS (NCT02893332)	Randomized Phase III SBRT + TKI vs. TKI	200	NSCLC (EGFR-mutant)	≤5	SBRT	PFS
NORTHSTAR (NCT03410043)	Randomized Phase II LAT + TKI	143	NSCLC (EGFR-mutant)	No limit	SBRT Surgery	PFS
CHESS (NCT03965468)	Single arm Phase II SBRT with 1st line chemo + IO	47	NSCLC	≤3	SBRT	OS
LONESTAR (NCT03391869)	Randomized Phase III LAT (to 3 lesions) + IO vs. IO	360	NSCLC	No limit	SBRT Surgery RFA	OS
NRG LU-002 (NCT03137771)	Randomized Phase II/III SBRT +MCT vs. MCT	400	NSCLC	≤3	SBRT	PFS (Phase II) OS (Phase III)
SARON (NCT02417662)	Randomized Phase III SBRT + SOC vs. SOC	340	NSCLC	≤3	SBRT	OS
SABR-COMET-3 (NCT03862911)	Randomized Phase III SBRT + SOC vs. SOC	297	Multiple	≤3	SBRT	OS
SABR-COMET-10	Randomized Phase III SBRT + SOC vs. SOC	159	Multiple	≤10	SBRT	OS

LAT: local ablative therapy; Sites: number of lesions, SBRT: stereotactic body radiotherapy; PFS: progression-free survival; OS: overall survival; IO: immunotherapy; MCT: maintenance chemotherapy; SOC: standard of care.
